# Oral status of HIV‐infected children aged 12 years or younger who attended a Paediatric Infectious Diseases Clinic in Cape Town

**DOI:** 10.1002/cre2.251

**Published:** 2019-11-13

**Authors:** Nadia Mohamed, Olorato Patience Mathiba, Riaan Mulder

**Affiliations:** ^1^ Department of Paediatric Dentistry University of the Western Cape Cape Town South Africa

**Keywords:** HIV/AIDS, paediatric, dental, caries, oral health

## Abstract

**Background:**

Children continue to suffer from the impact of the human immunodeficiency virus‐acquired immunodeficiency syndrome (HIV/AIDS) pandemic. In Cape Town, these children receive medical care including antiretroviral therapy from facilities like Tygerberg Hospital's Paediatric Infectious Diseases Clinic. HIV‐infected children may experience an increased caries experience when compared with their healthy peers.

**Aim:**

The aim of this study was to determine the oral health status of HIV‐infected children younger than 12 years receiving antiviral drugs at the Paediatric Infectious Diseases Clinic.

**Design:**

A cross‐sectional survey was conducted among children aged between 2 and 12 years presenting at this clinic. Caregivers were interviewed to obtain information regarding health seeking behaviour, oral hygiene practices and dietary habits. A single clinician undertook a standardized clinical intraoral examination according to the World Health Organization guidelines, with modifications.

**Results:**

Sixty‐six children were recruited. A high prevalence of dental caries (78.8%) and an unmet treatment need of 90.4% were recorded among the participants. Most children had never visited the dentist, and those who did had mainly received emergency dental care.

**Conclusion:**

The high prevalence of severe dental caries in this population highlights the need for oral health awareness and the inclusion of oral health care in the comprehensive care of children with HIV.

**Why this paper is important to paediatric dentists:**

- The study highlights the importance of collaborating with health professions outside of dentistry.
- Doctors and nurses are often the first health professionals to come into contact with children with special needs. They should therefore be made aware of the early signs of decay so that these patients can be referred for dental treatment timeously.
- Holistic management of children with special healthcare needs is essential to improve their overall well‐being.

## 1 INTRODUCTION

Since the discovery of the human immunodeficiency virus (HIV) in 1981, the virus has continued to wreak havoc globally with new infections continuing to surface and HIV‐related deaths still being registered. By the end of 2017, there were 36.9 million people living with HIV/AIDS worldwide (World Health Organisation, 2019). The impact of HIV has continued to be hard‐felt in Sub‐Saharan Africa, with the region accounting for 71% of the global HIV infections (UNAIDS HIV/AIDS gap report, 2014). South Africa on the other hand has an estimated 7.1 million people living with HIV; making it the country with the highest prevalence of people living with HIV/AIDS in the world (UNAIDS HIV and AIDS estimates, 2019).

Children constitute a vulnerable population as they continue to suffer the impact of the HIV/AIDS pandemic. The most recent report on HIV/AIDS in South African children between the ages of 0 and 14 years depicts close to 300,000 children living with HIV, that is, 3.9% of all HIV cases in South Africa (UNAIDS HIV and AIDS estimates, 2019). A study carried out in South Africa, which reported on the prevalence of HIV in different parts of the country, estimated a prevalence of 5.6% in children between the ages of 2 and 15 years in the Western Cape Province (South African National HIV Prevalence, Incidence and Behaviour Survey, 2012).

Higher risk of dental caries is a problem in children with HIV especially in the developing world. HIV‐infected children may experience an increased caries experience when compared with their healthy peers for various reasons (dos Santos et al., 2009; Howell, Palumbo, & Houpt, 1992). Immunocompromised children from resource‐limited populations (Naidoo & Chikte, 2004; Ramos‐Gomez, 2002) are particularly vulnerable. Higher caries risk has been attributed to socio‐economic factors such as poor access to medical resources and preventive measures (Ramos‐Gomez & Folayan, 2013). One study reported an increased caries rate in HIV‐infected children mainly due to the inappropriate dietary habits brought on by the failure to thrive (Madigan, Murray, Houpt, Catalanotto, & Feurman, 1996). Some studies have suggested that the caries experience in children is related to the acidic and sugary syrup medications consumed by these HIV patients, opportunistic infections and reduction in the salivary flow rate (dos Santos et al., 2009; Jetpurwala & Jain, 2011). An increased caries incidence has also been attributed to negligence on the caregiver's part concerning oral health and dietary habits of the child (Nokta, 2008).

Children living with HIV/AIDS in the city of Cape Town, Western Cape Province, receive medical care including antiretroviral drugs from various health care facilities. Tygerberg Hospital is one of these institutions with a Paediatric Infectious Diseases Clinic (PIDC) dedicated to providing care for children with HIV/AIDS. The hospital has approximately 300 children enrolled in their programme. The oral health status of children living with HIV/AIDS enrolled with the Tygerberg PIDC was unknown. The purpose of this study was therefore to conduct an oral health survey among HIV‐infected children younger than twelve years of age presenting at the Paediatric Infectious Disease Clinic at Tygerberg Hospital to determine the factors contributing to their oral health status.

The objectives of the study were to determine the following in the study population:

- the caries experience;
- the dental treatment need; and
- factors contributing to the oral health status.

## 2 MATERIALS AND METHODS

A cross‐sectional descriptive study was conducted between August 2015 and June 2016. Approval to conduct the study was sought from the University of the Western Cape Research Ethics Committee (Project Registration Number: 15/6/83). Permission to carry out the research at the PIDC was obtained from the relevant authorities at Tygerberg Hospital.

Even though children up to the age of 16 years are seen at this clinic, only children with a confirmed HIV‐positive status between 2 and 12 years of age were enrolled in the study, as the upper age limit for paediatric dental patients at the adjacent Tygerberg Oral Health Centre is 12 years of age. In order to be included in the study, children had to be in possession of a consent form signed by the legal guardian, providing permission for their child to be enrolled in the study. Children who did not comply with the clinical examination as well as those who were not in possession of a signed consent form were excluded from the sample. However, brief oral health advice was given to all the children and their caregivers.

A simple convenience sampling method was employed to choose candidates who fit the selection criteria. Children living with HIV/AIDS are seen at Tygerberg PIDC from Monday to Wednesday. On the days of the examination, all patients attending the outpatient clinic who were eligible for inclusion in the study were recruited. A statistician was consulted to assist with an estimation of the sample size. After doing a power calculation, 50 patients were found to be representative of the study population. A total number of 66 patients met the inclusion criteria and were recruited for the study. Participants were made aware of the study and given all the relevant information both verbally and in writing. Participation was entirely voluntary, with participants given the freedom to drop out of the study at any point in time. A written consent form was thoroughly explained and issued to each parent in English. In an effort to protect the identity and the confidentiality of the participants of this study, the patients' names were not recorded on the data capture sheet. Instead, the patients' medical record numbers were used for identification purposes.

The data collection process consisted of both quantitative and qualitative aspects. The former was used to capture the DMFT/dmft. The DMFT/dmft index has been used in several studies to record the caries experience. It is expressed as DMFT for permanent teeth and dmft for primary teeth (Klein & Knutson, 1938) and reported as a percentage of the total number of teeth present intraorally.

Socio‐demographic aspects of oral health were recorded through qualitative methods. Data collection was done through the use of a questionnaire and a clinical examination. A structured questionnaire adapted from the World Health Organization (WHO) Oral Health Surveys‐Basic Methods (2013) was administered orally through an interview conducted with the child and their legal guardian or caregiver. The interview was administered in English. The data captured from the interview included an inquiry on the child's oral hygiene practices, dietary habits and any past experiences of dental and orofacial ailments. A pilot study was carried out on 5 patients with the help of a co‐investigator (R. M.). The purpose of this process was to pilot the questionnaire and the adequacy of the data capture sheet. Unnecessary questions were omitted from the questionnaire and restructured where necessary.

A standardized clinical examination was carried out on all participants by the principal researcher (O. M.) who is a qualified dental practitioner. For each child, a basic examination pack consisting of an explorer probe, an intraoral mirror, gauze and a light‐emitting diode head‐light was used for the examination. To assess the dentition of the child, the WHO Oral Health Survey‐Basic Methods (2013) criteria were followed. Each tooth was examined using an intraoral mirror and an explorer probe. All the carious lesions, restorations and present/missing teeth were recorded. A data capture sheet was used to record the information obtained from the clinical assessment. The data capture sheet was generated by the principal researcher through an amalgamation of the basic charting format used in the Paediatric Dentistry Department at the University of the Western Cape and a form adapted from the WHO Oral Health Surveys‐Basic methods (2013). The data capture sheets for each patient enrolled in this study were kept safely in a locked cabinet in the Principal Researcher's office.

The data were entered into a Microsoft Excel 2010 spreadsheet. Statistical analysis was performed using the R‐project program (Statistical analysis with R Core Team (2013); (R: A language and environment for statistical computing. R Foundation for Statistical Computing, Vienna, Austria).

Descriptive statistical analysis was done for quantitative variables in the form of means, standard deviations (*SDs*) and 95% confidence intervals (CIs).

## 3 RESULTS

Out of the 66 children who were recruited for this study, 28 (42.4%) were female and 38 (57.6%) were male. All had one or more remaining deciduous teeth, except two study participants who had only permanent teeth (*n*=64). The overall caries experience in the primary teeth (dmft>0) was 78.1% (95% CI, 66.0–87.5), with minor differences between the two age and gender subgroups (Table 1).

**Table 1:** Caries experience in the primary dentition according to gender and age group (*n*=64)

Characteristic		dmft		
		dmft = 0	dmft > 0	Mean dmft ± *SD*
Gender	Female	7 (10.9%)	19 (29.7%)	5.46 ± 4.95
	Male	7 (10.9%)	31 (48.4%)	6.37 ± 4.55
Age group	2–6	10 (15.6%)	24 (37.5%)	5.88 ± 5.34
	7–12	4 (6.3%)	26 (40.6%)	6.13 ± 3.94

Thirty study participants had only deciduous teeth, and 36 had one or more permanent teeth. The caries experience (DMFT>0) was 41.7% (95% CI, 25.5–59.2), with minor differences between the two gender subgroups (Table 2).

**Table 2:** Caries experience in the permanent dentition according to gender (*n*=36)

Characteristic		DMFT		
		DMFT = 0	DMFT > 0	Mean ± *SD*
Gender	Female	11 (30.6%)	7 (19.4%)	0.89 ± 1.37
	Male	10 (27.8%)	8 (22.2%)	0.83 ± 1.20

Table 3 depicts the distribution of mean decayed (D‐/d), missing (M‐/m‐) and filled (F‐/f‐) teeth among the different age groups. The distribution of decayed, missing and filled deciduous and permanent teeth was 6.3 (*SD* 5.0) and demonstrated minor differences between the two age subgroups (Table 3).

**Table 3:** Distribution of decayed, missing and filled teeth according to the age group 2 to 6 years and 7 to 12 years

Age group	D‐/d‐	M‐/m‐	F‐/f‐	DMFT/dmft ± *SD*
2–6 years	5.12 ± 5.44	0.74 ± 2.08	0	5.97 ± 5.43
7–12 years	5.09 ± 4.28	1.38 ± 3.18	0.125 ± 0.71	6.63 ± 4.53
Overall DMFT‐/dmft‐				6.30 ± 4.99

According to the questionnaires completed by the parent or guardian, the most common frequencies were 60.6% (never experienced discomfort), 60.6% (never visited the dentist), 13.6% (experienced discomfort), 42.4% (brushed once per day), 60.6% (made use of adult toothpaste). No problems were reported by 67.9% of study participants (Table 4). The consumption of carbohydrates related to dietary habits was relatively high (Table 5).

**Table 4:** Frequency of oral hygiene practices according to questionnaire completed by parent (*n*=44) or guardian (*n*=22)

Variable	Categories	Frequency *n* (%)
Discomfort in the mouth	Often	3 (4.5)
	Occasionally	15 (22.7)
	Rarely	1 (1.5)
	Never	40 (60.6)
	Don't Know	7 (10.6)
Previous dental visits	Once	11 (16.7)
	More than once	3 (3.5)
	None in the past 12 months	7 (10.6)
	Never	40 (60.6)
	Don't know	5 (7.6)
Reason for visit	Discomfort	9 (13.6)
	Check‐up	2 (3.0)
	Follow‐up	2 (3.0)
	Don't know	2 (3.0)
	N/A	51 (77.3)
Frequency of tooth brushing	Never	6 (9.1)
	Once a day	28 (42.4)
	Two or more times a day	26 (39.4)
	Occasionally	6 (9.1)
Toothpaste used	Adult toothpaste	40 (60.6)
	Baby toothpaste	19 (28.8)
	None	7 (10.6)
Problems experienced	Aesthetics	6 (9)
	Chewing	8 (12.1)
	Sleep disturbance	1 (1.5)
	School attendance	1 (1.5)
	Don't know	4 (6.1)
	None	46 (69.7)

**Table 5:** Frequency of dietary habits according to questionnaire completed by parent (*n*=44) or guardian (*n*=22)

Variable	Categories	Frequency *n* (%)
Fresh fruit	Never	1 (1.5)
	Everyday	1 (1.5)
	Once a week	1 (1.5)
	Several times a week	41 (62.1)
	Several times a month	22 (33.3)
Sugary snacks	Never	1 (1.5)
	Everyday	22 (33.3)
	Several times a day	36 (54.5)
	Several times a week	1 (1.5)
	Several times a month	6 (9.1)
Sugary drinks	Never	1 (1.5)
	Everyday	28 (42.4)
	Several times a day	20 (30.3)
	Several times a week	17 (25.8)
Candy/Sweets	Never	3 (4.5)
	Several times a day	2 (3.0)
	Several times a week	15 (22.7)
	Several times a month	46 (69.7)
Coffee/tea with sugar	Never	2 (3.0)
	Everyday	32 (48.5)
	Several times a day	1 (1.5)
	Several times a week	9 (13.6)
	Several times a month	22 (33.3)

## 4 DISCUSSION

A high proportion of children in this study presented with a severe pattern of dental caries. An overall prevalence of 78.8% (95% CI, 67.0–87.9) was recorded among the study population, that is in 52 of 66 participants. In the primary dentition, a prevalence of 78.1% (95% CI, 66.0–87.5) was found; significantly higher than the 41.7% (95% CI, 25.2–59.2) found in the permanent teeth. The results of this study corroborate reports of a high caries experience amongst HIV‐infected children, especially in the primary dentition (Howell et al., 1992; Nabbanja, Gitta, Peterson, & Rwenyonyi, 2013; Yengopal, Kolisa, Thekiso, & Molefe, 2016). Studies have reported caries prevalences ranging from 40% to 86% in the primary dentition (Beena, 2011; Meless, Ba, Faye, et al., 2014; Nabbanja et al., 2013; Rwenyonyi et al., 2011).

Looking at the severity of the caries experience in the deciduous dentition, the mean dmft was 6.0 ± 4.70 with no significant difference between males (6.37 ± 4.55) and females (5.46 ± 4.95). There was no significant difference in the mean dmft of the two age groups, that is, 2 to 6 years and 7 to 12 years. In the scientific literature, the reported mean dmft ranges between 1.5 and 11.8 (Cerqueira, Portela, & Pomarico, 2010; dos Santos et al., 2009; Madigan et al., 1996; Meless et al., 2014). Contrary to the findings of the current study, another study found the dmft in children with perinatally acquired HIV was significantly lower and comparable to that of normal children (Sahana, Krishnappa, & Krishnappa, 2013). A meta‐analysis concluded that even though studies reported a high dmft, there was no significant association between the caries experience and HIV‐infection (Oliveira et al., 2015).

In the permanent dentition, the mean DMFT for the sample population was 0.86 ± 1.29. There was no significant variation between girls (0.89 ± 1.37) and boys (0.83 ± 1.20). Several studies have reported mean DMFT ranging from 0.5 to 4.0 (Cerqueira et al., 2010; dos Santos et al., 2009; Madigan et al., 1996; Meless et al., 2014; Sahana et al., 2013). The results of the current study highlighted an overall low caries experience (mean DMFT) in the permanent dentition. Similarly, another study found that the data on the caries experience in the permanent dentition although insufficient, revealed a low mean DMFT (Oliveira et al., 2015).

The older age group, 7 to 12 years had a higher mean decayed (D‐/d‐) component. This substantiates conclusions that older children have more decayed teeth compared with the younger children because their teeth are exposed to environmental risk factors for longer (Ferraro & Vieira, 2010; Wang, Willing, Marazita, et al., 2012). In the present study, decayed teeth accounted for 82.2% of the total number of DMFT/dmft. According to literature the F‐/f‐ component is rarely reported in children and if reported, the numbers are usually low (Nabbanja et al., 2013; Rwenyonyi et al., 2011). In the present study, only one child had received restorative treatment, represented by a low mean filled index (F‐/f‐) of 0.125. The overwhelming majority of children received extractions for the treatment of dental caries. The undesirable consequences of premature loss of deciduous teeth have been thoroughly discussed in the literature. Early loss of primary teeth may predispose one to crowding and malposition of permanent teeth resulting from loss of arch length (Ahamed et al., 2012).

Of the 52 children (78.8%) with dental caries experience, 47 had untreated carious teeth. This value, described as the “Unmet Treatment Need (UTN),” is calculated by dividing the percentage of untreated caries by the caries prevalence (Van Wyk & Van Wyk, 2004). In the current study, an UTN of 90.4% was found among participants. This correlates to the 92% UTN among 4‐to‐5‐year‐old children reported by Van Wyk and Van Wyk (Van Wyk & Van Wyk, 2004). Two studies conducted by the same authors in 2012 investigated the caries experience among children living with HIV in South Africa (Joosab, Yengopal, & Nqcobo, 2012). They found prevalences of 62.9% and 70.9% and mean dmft indices of 4.2 and 5.1, respectively. The UTN among children in this study ranged from 87.8 to 100%. The report on the National Children's Oral Health Survey (Van Wyk & Van Wyk, 2004) concluded that approximately 45 to 60% of children in South Africa required treatment for dental caries. In the Western Cape Province, this UTN was reported to be as high as 80% (Ramphoma, 2016; Singh, 2011). The results of the current study are therefore no different from other findings with regards the caries experience among children in South Africa.

Medically compromised children who are on long‐term medications are generally classified as having a high caries risk (Foster, 2005). Children living with HIV have been found to be susceptible to dental caries (Leão, Ribeiro, Carvalho, Frezzini, & Porter, 2009). Studies have established a direct relationship between caries risk and HIV, particularly with respect to the potentially cariogenic and xerostomic antiretroviral medication (Nittayananta, Chanowanna, & Winn, 2010; Nittayanata, 2016; Oliveira et al., 2015). Other factors implicated in the increased prevalence of dental caries among children with HIV include diminished flow of saliva and a reduction in salivary antibodies (dos Santos et al., 2009; Oliveira et al., 2015). Due to the absence of an HIV‐negative control group in this study, a direct relationship between the HIV infection and caries prevalence/severity could not be established. This is therefore a potential area of research that should be explored in future clinical studies.

Control of oral disease is often dependent upon several environmental factors such as dietary factors, family factors, behavioural factors and access to oral health services (Hashim, Thomson, Ayer, Lewsey, & Awad, 2006). The family and social environment include the child's caretaker, the number of siblings in a household as well as household crowding (Petersen, Bourgeois, Ogawa, Estupinan‐Day, & Ndiaye, 2005; Wang et al., 2012). In this study, 21.2% of children were under the care of an institution or foster care, categorised as “other.” The institutionalized children were orphaned and had concomitant ailments. Children under the care of a relative were cared for by a grandparent, an aunt or an uncle. Most of the children hailed from poor households. The association between poor oral health in children and low socioeconomic status of the family has been widely documented in the literature (Castilho, Mialhe, Barbosa, de, & Puppin‐Rontani, 2013; Petersen et al., 2005).

Access to oral health care is a crucial factor in the prevention and management of dental caries (Singh, 2011). In the present study, out of the entire sample, only 21.2% had had a dental visit in the previous year; 60.6% had never been to the dentist nor had a dental check‐up. Negligence towards oral health has been shown to be among the leading factors related to the development and progression of dental caries (Castilho et al., 2013). In addition, oral hygiene practices as well as exposure to fluoride have been shown to play a key role as protective factors in minimising the caries risk (Yengopal et al., 2016). In the present study, 42.4% of the sample admitted to brushing their teeth only once a day, and 18.2% either brushed occasionally or had never brushed. These inadequate tooth brushing practices could be linked to the high caries experience.

The impact of diet and nutrition, especially the role played by refined sugary substances on the development and progression of dental caries has been widely discussed in the literature (Rwenyonyi et al., 2011). The dietary habits of children were explored, particularly the consumption of sugary foods. In this study, a large proportion of children consumed sugary snacks and drinks several times a day. The frequency of these sugar attacks was exceptionally high.

The present study was aimed at highlighting the oral health status among children living with HIV, with regard to dental caries. A significant number of children presented with dental caries, the majority of which were untreated. The lack of restorative treatment was quite evident. Among the several factors that were relevant to the increased prevalence of dental caries in children with HIV was lack of awareness regarding oral health issues and inadequate access to oral health services.

## 5 LIMITATIONS OF THE STUDY

A potential limitation of this study was the small sample size present at the PIDC. A larger sample size would have been more reflective of the oral health status of children in South Africa. However, at some point during the data collection period, the same children were seen, thus depicting sample saturation. Future studies should also be carried out in other provinces in South Africa.

## 6 CONCLUSION

This study highlights the need for oral health awareness and access to oral health services for children with HIV. Most of the caregivers in this study were unaware of services offered at Tygerberg Dental Faculty, University of the Western Cape. The need for restorative treatment can never be over emphasized. Tygerberg Dental Faculty has a fully functional paediatric department offering comprehensive restorative treatment. The notion that primary teeth are just temporary teeth and therefore dental care is not required still resonates among most parents and caretakers.

This data also reflects the need for a collaborative effort between the PIDC and paediatric dental clinics to provide oral health services to children with HIV. Doctors and nurses are often the first health professionals that come into contact with these patients. Training these professionals to recognize the early signs of dental caries will therefore go a long way to providing appropriate holistic care for these patients. This can only be facilitated by improved screening and referral processes between the medical and dental professions.
